# Signs of impending rupture in abdominal aortic and iliac artery aneurysms by computed tomography: Outcomes in 41 patients

**DOI:** 10.6061/clinics/2021/e2455

**Published:** 2021-03-01

**Authors:** Bruno Fabricio Feio Antunes, Adriano Tachibana, Cynthia de Almeida Mendes, Lucas Lembrança, Marcela Juliano Silva, Marcelo Passos Teivelis, Nelson Wolosker

**Affiliations:** I Departamento de Cirurgia Vascular, Hospital Israelita Albert Einstein, Sao Paulo, SP, BR; II Departamento de Radiologia, Hospital Israelita Albert Einstein, Sao Paulo, SP, BR

**Keywords:** Aortic Aneurysm, Abdominal, Computed Tomography Angiography, Aortic Aneurysm, Rupture, Ruptured Aneurysm

## Abstract

**OBJECTIVES:**

This study aimed to determine the prevalence of signs of impending rupture (SIR) in asymptomatic patients with abdominal aortic and iliac artery aneurysms, and to evaluate whether these signs were associated with rupture in asymptomatic patients.

**METHODS:**

This was a retrospective study of patients with abdominal aortic and iliac artery aneurysms identified on computed tomography (CT) over a 10-year period in a single center. The CT scans were reviewed by two reviewers, and patients with SIR were assigned to one of three groups: (1) early symptomatic (ES), (2) late symptomatic (LS), and (3) always asymptomatic (AA). The four main SIR described in the literature were investigated: 1) crescent sign, 2) focal wall discontinuity of circumferential calcifications, 3) aortic bulges or blebs, and 4) aortic draping.

**RESULTS:**

From a total of 759 aortic and iliac aneurysm reports on 2226 CT scans, we identified 41 patients with at least one SIR, and a prevalence of 4.14% in asymptomatic patients. Focal wall discontinuity of circumferential calcifications was the most common sign, and it was present in 46.3% of these patients (19/41); among these, 26 were repaired (ES: 9, LS: 2, AA: 15). Eleven asymptomatic patients underwent follow-up CT. The aneurysm increased in size in 6 of the 11 (54.5%) patients, and three ruptured (all with discontinuity of calcifications), one of which had no increase in diameter.

**CONCLUSIONS:**

The presence of focal wall discontinuity of circumferential calcifications was the most common SIR. There was a prevalence of all signs in less than 5% of asymptomatic patients. In unrepaired patients, the signs could be observed on follow-up CT scans with an increase in aneurysm size, indicating that the presence of SIR alone in the absence of other clinical factors or aneurysm characteristics is an insufficient indication for surgery.

## INTRODUCTION

The rupture of an abdominal aortic aneurysm (AAA) is one of the most dramatic emergencies in medicine; only approximately 50% of patients with ruptured AAAs are alive by the time they reach the hospital, among whom, up to 50% do not survive repair (1).

Imaging is essential for the early detection and size measurement of AAAs. Elective repair of the aneurysm is indicated for rapidly expanding aneurysms or for those that exceed 5.5 cm in diameter in men and 5.0 cm in women as prophylaxis against rupture; the 30-day mortality ranges from 0.9% to 4.8% depending on the type of repair and center experience (2). Previous reports have concluded that screening for AAA reduces the incidence of aneurysm rupture and AAA-related mortality (3).

Computed tomography (CT) angiography has become an increasingly useful modality for the diagnosis of AAAs and surveillance of the aortoiliac axis, both in elective surgery situations, to assess the aneurysm with more comprehensive anatomical details, and for patients in whom rupture is suspected (4-6). Additionally, CT angiography incidentally detects aneurysms on exams performed for other purposes.

Interestingly, four signs on CT scans have been suggested as indicators of an increased risk of AAA rupture. These “signs of impeding rupture” (SIR) include the following: 1) crescent sign (4,5,7-9), 2) focal wall discontinuity of circumferential calcifications (5), 3) aortic bulges or blebs (10), and 4) aortic draping (8).

To the best of our knowledge, there are only 16 studies that have directly examined these signs of ‘impending’ rupture, including eight review studies (4,6-8,11-14) six case series (5,10,15-18), and two case reports (19,20), with most discussing only one specific sign.

The aim of this study was to determine the prevalence of SIR in asymptomatic patients with abdominal aortic and iliac artery aneurysms who underwent CT of the abdomen and pelvis at a single center. In addition, we sought to evaluate whether these signs were associated with rupture in asymptomatic patients, and to determine the mean time until rupture for each sign.

## METHODS

This was a retrospective study for which the waiver of informed consent was approved by the Institutional Ethics Committee. We performed a word search of our comprehensive computer database containing all reports of CT scans of the abdomen and pelvis performed in symptomatic and asymptomatic patients with aortic and/or iliac artery aneurysms at the Albert Einstein Jewish Hospital (HIAE), São Paulo, Brazil between October 2007 and October 2017 using the keyword “aneurysm.” All CT scan reports were evaluated for the presence of imaging features, which may herald instability in the aortoiliac axis. CT scan reports with signs of aneurysm instability were retrospectively reviewed by the consensus opinion of two reviewers, one experienced vascular surgeon and one radiologist. Specifically, for the analysis of focal wall discontinuity of circumferential calcifications, CT scans were compared with previous imaging examinations that were available in our record retrieval system.

Aortic and iliac artery aneurysms were defined as a dilation of the aorta and iliac arteries to a diameter greater than 3.0 cm and 1.8 cm, respectively (21). Symptomatic patients were defined as those with sudden-onset abdominal and/or low back pain that may radiate to the flank or groin (22).

Patients with ruptured aneurysms with reported contrast extravasation and/or abdominal hematoma independent of clinical symptoms, mycotic aneurysms, anastomotic aneurysms, visceral aneurysms, or extremity aneurysms were excluded from the study.

**Figure 1:**
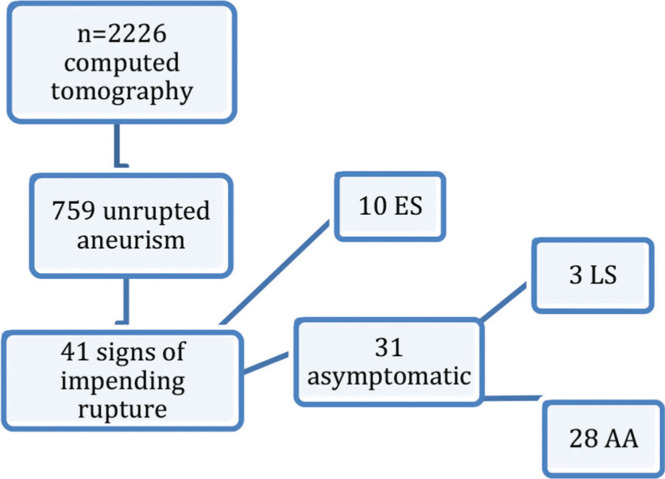
Flowchart with the distribution of patients by group.

Patients were assigned to one of three groups as follows (Figure 1): The first group, hereafter referred to as early symptomatic (ES), comprised patients presenting with abdominal and/or low back pain who were referred for CT examination; the second group, referred to as late symptomatic (LS), included patients who were initially asymptomatic and underwent a CT scan for purposes other than the aneurysm, but later developed symptoms that prompted another CT scan, and the third group, hereafter referred to as always asymptomatic (AA), comprised patients who underwent imaging examinations for complaints unrelated to the aneurysm and who never manifested aneurysm-related symptoms. The data collected from the medical records included:

1. Demographic data (age, sex)
2. Clinical signs and symptoms that prompted the CT examination (we discriminated against exams performed for monitoring symptomatic aneurysms and those requested for other purposes)
3. Decision frequency: surgical, endovascular, or clinical treatment
4. Comorbid conditions (smoking, systemic arterial hypertension [SAH], diabetes, and dyslipidemia)
5. Operative findings (in patients who underwent surgical intervention)
6. Incidence of aneurysm rupture in patients who did not undergo surgical repair, and the clinical manifestation of ruptured aneurysm in these patients
7. Time interval (days) from CT examination to surgical intervention, or time interval between the index and follow-up CT scans for the determination of time until rupture

### Data analysis

Categorical variables are reported as absolute and relative frequencies. Age is reported as the mean±standard deviation, and the other quantitative variables are reported as median and first and third quartiles. Comparisons between two groups were performed using the Mann-Whitney *U* test for quantitative variables because the data did not meet the assumption of normality. The Kruskal-Wallis test was used for comparisons between the three groups and analysis of variance (ANOVA) was used for age. Categorical variables were compared between the groups using Fisher’s exact tests (23) because the number of expected values was less than five for all comparisons.

The analyses were performed using the R Core Team and IBM SPSS software packages. The level of significance was defined as *p*<0.05.

## RESULTS

We retrospectively reviewed 2226 CT scans performed between October 2007 and October 2017 with the word “aneurysm” included in the reports. The record retrieval system identified 759 patients with unruptured abdominal aortic aneurysms and/or iliac artery aneurysms, of which 648 were infrarenal or juxtarenal aortic aneurysms and 111 were iliac artery aneurysms without aortic involvement.

Forty-one CT scan reports showed signs of aneurysm instability, including 39 aneurysms in the infrarenal or juxtarenal aorta and two in the iliac artery only. Ten patients were symptomatic on presentation, and 31/749 (4.14%) patients were asymptomatic with at least one CT SIR.

The demographic data and characteristics of the sample are summarized in Table 1. Most patients across the three groups were male, in the 7^th^ and 8^th^ decades of life, smokers, with a history of hypertension and dyslipidemia.

The diameter of the aortic aneurysms ranged from 3.0-12.0 cm and 53.7% were ≥5.5 cm. There was no significant difference in the median size of the aortic aneurysms between the groups. Ten (24.4%) of the 41 patients had iliac aneurysms with diameters ranging from 2.0-5.9 cm. Two patients had isolated iliac artery aneurysms with SIR; one underwent surgical repair without intraoperative signs of rupture, and the other underwent a follow-up CT scan 392 days after the index CT scan and was observed for a further 515 days without rupture.

The sizes of the aortic and iliac aneurysms are presented in Table 2.

Group comparisons for clinical characteristics and SIR-Focal wall discontinuity of circumferential calcifications was the most common CT imaging SIR, and was present in 19 patients (46.3%). There was no statistically significant difference in the prevalence of SIR between the three groups.

The SIR and the patients with more than one sign are listed in Table 3. Nine (22.0%) of the 41 patients had two or more SIRs, one of whom became symptomatic. There were no significant differences in the rupture or mortality rates between patients with more than one concurrent SIR and patients with only one SIR, even among those who underwent surgery, and no difference in the size of their aneurysms was detected.

Group comparisons by surgical status The majority of patients in all groups underwent surgical repair. There use of open and endovascular repair was similar in the symptomatic groups (ES and LS), and there was a trend towards endovascular repair in AA patients; however, no significant difference in the type of repair was found between the groups (Table 4). No pathologic reports were available, and the operative reports contained no information about structural abnormalities in the arterial wall in patients who underwent open aneurysm repair, except in cases of rupture with retroperitoneal hematoma. No specific mention was made to other aortic wall abnormalities (*e.g.*, infection).

**Figure 2:**
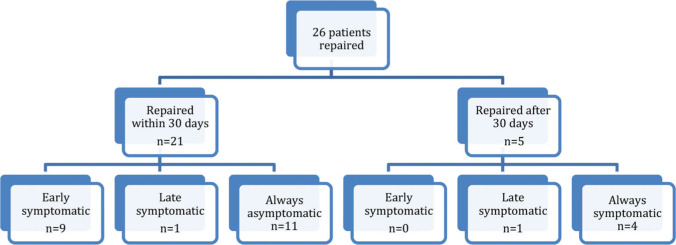
Flowchart of patients who underwent aneurysm repair by symptom status.

Twenty-six of 41 patients underwent surgical repair, most of whom were in the AA group and were repaired within 30 days of CT examination (Figure 2).

The surgical details, time interval between the CT scan and surgical repair, and the number of in-hospital deaths are presented in Table 4. The time interval between the index CT scan and surgery ranged from 0-1961 days (∼5.4 years) and was significantly longer among ES patients (*p*=0.001), most of whom underwent repair on the same day.

The mean time until rupture was 358.6 days (range, 3-2616 days) for patients with focal wall discontinuity of circumferential calcifications, 335.8 days (range, 3-1961 days) for patients with crescent sign, 159 days (range, 10-716 days) for patients with aortic bulges or blebs, and 3 days (range, 1-5 days) for patients with draped aorta.

Nine of 10 patients in the ES group underwent surgical repair, two of whom had intraoperative signs of rupture and died; both had aneurysms >10 cm in diameter, one had focal wall discontinuity of circumferential calcifications and the other had a draped aorta sign. Most patients (5) underwent emergency repair on the same day (range, 0-4 days), including two patients with intraoperative signs of rupture. The only patient in the ES group who did not undergo surgery was an elderly patient with multiple comorbidities whose family members opted for palliative care; as expected, the patient died during the same hospitalization for aneurysm rupture.

Three of the 31 asymptomatic patients developed symptoms (LS) at 5, 95, and 479 days after the index CT scan. The patient who manifested symptoms at 95 days with a CT diagnosis of rupture was an elderly patient with multiple comorbidities who received palliative care and died within 72h of rupture. The other two patients underwent surgery and survived. All LS patients had focal wall discontinuity of circumferential calcifications, and one patient had a concurrent crescent sign. Fifteen of 28 AA patients underwent repair; the vast majority (11) were repaired within 30 days of the CT scan, one underwent surgery on the same day, four underwent surgery during the same hospitalization. The remaining 13 patients never underwent repair and were observed from 0 to 1961 days without rupture.

Nineteen of the 26 patients who underwent surgical repair were male, six patients had aneurysms <5.5 cm in diameter, and only one of these six patients was symptomatic. Only one of the seven women who underwent surgery had an aneurysm <5.0 cm, and the patient was asymptomatic.

Excluding the 10 ES patients, 11/31 underwent a follow-up CT scan (Table 5), three of whom were LS and eight were AA.

The aneurysm size in the 11 patients with follow-up CT scans ranged from 3.0-10.0 cm on follow-up CT, and the time interval between the index and follow-up CT examinations ranged from 5-1961 days. Aneurysms increased in 6 (54.5%) of the 11 patients, with a mean increase of 1.71 cm.

Six of the 11 patients with follow-up CT had focal wall discontinuity of circumferential calcifications, three had aortic bulges or blebs, two had the crescent sign, and none had a draped aorta. There was no evidence of a difference in the SIR between the groups on follow-up CT, all SIRs could be seen, and no new signs were observed.

Four of the 26 patients who underwent surgical repair had rupture (*i.e.*, signs of rupture were noted during surgery). Of these four, two were ES patients with the draped aorta sign and focal wall discontinuity of circumferential calcifications, one was an LS patient with the crescent sign and discontinuous wall, and one was an AA patient with a crescent sign and an aneurysm that was 5.2 cm in size that increased to 8.6 cm on follow-up CT at 1961 days.

## DISCUSSION

Awareness and recognition of the imaging findings associated with impending rupture are a diagnostic challenges with important repercussions on treatment promptness and prognosis.

**Figure 3:**
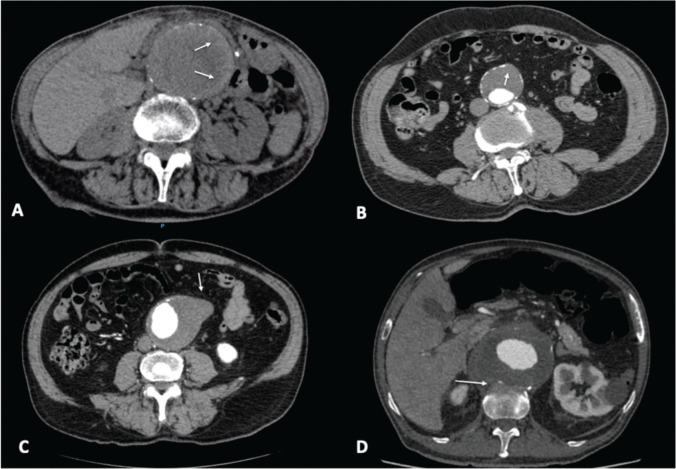
Axial abdominal CT images showing infrarenal abdominal aortic aneurysms with SIR (white arrows). A: Crescent sign. B: Focal wall discontinuity of circumferential calcifications. C: Aortic bulges or blebs. D: Draped aorta sign.

CT imaging findings referred to as “signs of aneurysm instability” or “signs of imminent rupture” use strong terminology, which tends to influence treatment decision-making, and are taken to predict short-term aneurysm rupture. A peripheral crescent sign (Figure 3) is defined as increased attenuation within the thrombus of the AAA (4,7-9). The most common imaging manifestation of the rupture process, the crescent sign represents the dissection of blood into the thrombus (10) and was initially described in non-contrast enhanced CT scans. Since dissection in the thrombus directly communicates with the lumen, this sign is best appreciated with contrast agent in the arterial phase in scans (sign of thrombus fissuration).

Many aneurysms are lined with circumferential wall calcifications. In impending or complete AAA rupture, a focal wall discontinuity of circumferential calcifications can be seen (Figure 3), indicating the rupture site, which is most commonly observed on the posterolateral wall (5).

Aortic bulges or blebs (Figure 3) are impeding rupture sites that can also be seen as focal bulging of the aneurysm wall (aortic bleb). This sign corresponds histologically to inflammatory changes and focal thinning of elastic fibers associated with impending rupture (10).

The draped aorta sign (Figure 3) can be seen in contained AAA ruptures when the rupture site is posterior and sealed by the adjacent vertebral body. The posterior wall of the aorta moves to the anterior surface of the vertebra, following which, normal fat planes between the aneurysm and vertebra are lost. A draped aorta is a characteristic sign of rupture because erosion secondary to rupture leads to inflammation and spreading of arterial wall disruption, which may cause rupture in nearby sites by contiguity (5).

In our population, excluding the 10 patients who were symptomatic on presentation with pain or signs of hemodynamic instability, the prevalence of SIR in asymptomatic patients was 4.14%, a previously unreported figure for AAAs. The patients in the three groups (ES, LS, and AA) had similar clinical characteristics, except for a lack of documented history of SAH in LS patients. Due to the retrospective nature of our study and limitations such as incomplete documentation, missing charts, or unrecorded information, we believe that this difference was due to a data error rather than a different rupture prognosis for non-hypertensive patients.

Although our patient population was relatively small, to the best of our knowledge, this is the largest sample of patients with aortic aneurysms with SIR in the literature. Boules et al. (8) identified 39 patients with aortic aneurysms and CT SIR, including 26 infrarenal, two suprarenal, and 11 thoracoabdominal aneurysms. Our study focused on juxtarenal and infrarenal aneurysms; hence, our sample included 39 infrarenal and juxtarenal aortic aneurysms and two iliac artery aneurysms without aortic involvement. We cannot explain why isolated iliac aneurysms were relatively common in our cohort (>10%), while SIRs were not.

In our study, a focal wall discontinuity of circumferential calcifications was the most common imaging SIR (46.3%). Calcifications may arise both in the mural thrombus and the aneurysmal wall. However, even though these calcifications are observed in up to 24% of aneurysms with mural thrombus, they are not directly associated with rupture (11). A discontinuity of calcifications is a cause of concern for aneurysm disruption (24). Found in 8% of ruptured aneurysms, this sign is most useful when prior intact circumferential calcified walls have been documented on previous CT scans for comparison, as well as an increased aneurysmal sac (4). In our study, a focal wall discontinuity of circumferential calcifications was the most common sign detected in patients who died, and was most commonly associated with patients who were asymptomatic on the index CT scan and only developed symptoms later. However, as the number of patients in our sample was small and there were few deaths, this trend did not reach statistical significance.

Patients with aortic bulges or blebs and a draped aorta sign did not become symptomatic later. A draped aorta sign is considered an indication of contained rupture by many authors. However, of the four AA patients with the draped aorta sign in our sample, two showed no signs of rupture when they underwent surgery, and the other two had no index CT scan or reports of rupture or surgery in another service described in their medical records. The small number of patients precludes a definitive conclusion that these signs are any less “dangerous” than a focal wall discontinuity of circumferential calcifications, but this finding may indicate a trend that warrants further investigation in other patient populations.

Boules et al. (8) reported that the crescent sign was present in more than two thirds of all SIRs (symptomatic and asymptomatic), and was the most common finding of impending rupture in their study. We are uncertain of the reason for this difference, and although the authors do not provide evidence of why one sign may be more common than others, we believe that it is due to an equal likelihood of any sign arising.

The crescent sign, despite being the second most common sign (present in 13 cases), was not commonly observed in late symptomatic patients but was found in one AA patient with intraoperative signs of rupture. This case warrants further discussion; the patient was asymptomatic but was diagnosed as ruptured intraoperatively during endovascular surgical repair. The only finding on follow-up CT was the crescent sign (growth >3 cm between the index CT scan and follow-up CT examination at 1961 days). We were unable to establish if the patient ruptured between the CT scan and surgery, even without any documented symptoms, or if there were intraoperative complications that led to contrast extravasation.

All three patients who were completely asymptomatic on the index CT scan and only developed symptoms later had a focal wall discontinuity of circumferential calcifications, and one also had a crescent sign. The fact that one of two patients with only a focal wall discontinuity of circumferential calcifications developed symptoms only 5 days after the index CT scan is highly indicative of aneurysm instability, but this was an isolated event in our patient population.

Only 63.4% of patients underwent surgical intervention despite the presence of SIR, and those who had never been repaired had long rupture-free survival times of up to 2616 days. These findings suggest that these signs may be of little use in predicting rupture and indication for surgery.

The time until rupture in asymptomatic patients, that is, the time interval between the index CT scan and surgical repair or medical record without evidence of rupture or treatment (follow-up CT or other abdominal imaging examinations) in patients with LS and AA also provides invaluable information. These data suggest two opposing possibilities: from one point of view, if most aneurysms rupture within a short period of time (*i.e.*, 1 week), then SIR has predictive value for AAA rupture; however, if aneurysms are unruptured 5 years after the sign is detected, they can be considered as poor predictors of rupture.

The median follow-up of approximately 1 year at which patients remained asymptomatic, either because they were never repaired or were repaired while still asymptomatic, suggests that observation rather than immediate intervention is a relatively safe approach for most patients with SIR. These patients can then be scheduled for elective repair and receive proper preoperative management and aneurysm repair if the aneurysm size indicates the need for surgical management. Moreover, in cases where surgery is refused, either because the aneurysm diameter is below the threshold for surgery or other reasons, patients should always be advised to seek an emergency service if any symptoms arise.

Our study has some limitations. First, as this was a retrospective study conducted at a hospital with locum tenens physicians, the patient population was small and did not receive standardized management. However, despite this limitation, the study provides real-world evidence data. Another possible limitation of retrospective chart review studies is incomplete documentation, although we believe that no relevant information was lost for the more important outcomes.

The limited number of patients with available follow-up CT data (n=11) precluded statistical significance between the groups since most patients were repaired. However, this was expected given that the presence of SIR is still a cause for concern for the surgical team, the patient, and the patient’s family, and is normally taken to herald imminent rupture. Studies that have objectively followed patients with any SIR are essential, and our study makes important contributions to AAA treatment and prognosis.

To date, no study has been able to establish the real clinical significance of SIR, typically because previous studies were single-center studies with small samples. A multicenter study could increase the number of cases and follow-up to improve statistical power. While such a study has not yet been conducted, our data suggest a less relevant role for SIR.

## CONCLUSIONS

Less than 5% of asymptomatic patients with abdominal aortic and iliac artery aneurysms had any CT SIR. The presence of focal wall discontinuity of circumferential calcifications was the most common SIR. The time to rupture for most signs was longer than 100 days. In unrepaired patients, the signs could be seen on follow-up CT scans with an increase in aneurysm size, indicating that the presence of SIR alone in the absence of other clinical factors or aneurysm characteristics is an insufficient indication for surgery.

## AUTHOR CONTRIBUTIONS

Antunes BFF, Tachibana A, and Silva MJ helped to collect the data. Antunes BFF, Lembrança L and Teivelis MP analyzed the data. Antunes BFF, Mendes CA, Lembrança L and Teivelis MP helped to write the manuscript. Antunes BFF, Silva MJ, Lembrança L and Wolosker N interpreted the patient data and reviewed the manuscript. All authors approved the final version of the manuscript and are accountable for it.

## Figures and Tables

**Table 1:** Clinical characteristics of the sample by group.

	Initial symptomatic (10)	Late symptomatic (3)	Always asymptomatic (28)	Total (41)	*p*-value
Age					
Mean (SD)	73.1 (8.4)	79.7 (15.6)	77.6 (9.9)	76.6 (9.9)	0.419^A^
Sex					
Male	6 (60.0%)	1 (33.3%)	21 (75.0%)	28 (68.3%)	0.209
Female	4 (40.0%)	2 (66.7%)	7 (25.0%)	13 (31.7%)	
Smoker	7 (70.0%)	2 (66.7%)	12 (42.9%)	21 (51.2%)	0.392
Hypertension	8 (80.0%)	0 (0.0%)	23 (82.1%)	31 (75.6%)	0.020
Dyslipidemia	6 (60.0%)	0 (0.0%)	18 (64.3%)	24 (58.5%)	0.134
Diabetes	2 (20.0%)	1 (33.3%)	5 (17.9%)	8 (19.5%)	0.823

A: Comparison by ANOVA. Other comparisons by Fisher's exact test. SD: Standard deviation.

**Table 2:** Sizes of aortic and iliac aneurysms.

	Initial symptomatic (10)	Late symptomatic (3)	Always asymptomatic (28)	Total (41)	*p*-value
Size of aortic aneurysm					
Median (1^st^ Q, 3^rd^ Q)	57 (55, 72)	55 (30, 71)	52 (47, 63)	55 (47, 63)	0.527^K^
Minimum-maximum (n)	35-120 (10)	30-71 (3)	36-95 (28)	30-120 (41)	
Size of aortic aneurysms (mm)					
<45	2 (20.0%)	1 (33.3%)	5 (17.9%)	8 (19.5%)	0.063
45 to 54	0 (0.0%)	0 (0.0%)	11 (39.3%)	11 (26.8%)	
≥54	8 (80.0%)	2 (66.7%)	12 (42.9%)	22 (53.7%)	
Total	10 (100.0%)	3 (100.0%)	28 (100.0%)	41 (100.0%)	
Size of iliac aneurysm (mm)	3 (30.0%)	1 (33.3%)	6 (21.4%)	10 (24.4%)	0.606
Size of the iliac aneurysm					
Median (1^st^ Q, 3^rd^ Q)	47 (20, 59)	44 (44, 44)	33 (23, 35)	35 (23, 47)	0.623^K^
Minimum-maximum (n)	20-59 (3)	44-44 (1)	20-50 (6)	20-59 (10)	

K: Kruskal-Wallis test. Other comparisons were performed using Fisher’s exact tests. 1^st^ Q: First quartile, 3^rd^ Q: Third quartile.

**Table 3:** Signs observed by group.

	Initial symptomatic (10)	Late symptomatic (3)	Always asymptomatic (28)	Total (41)	*p*-value
Crescent sign	2 (20.0%)	1 (33.3%)	10 (35.7%)	13 (31.7%)	0.748
Focal wall discontinuity	4 (40.0%)	3 (100.0%)	12 (42.9%)	19 (46.3%)	0.206
Aortic bulges or blebs	2 (20.0%)	0 (0.0%)	10 (35.7%)	12 (29.3%)	0.458
Draped aorta	2 (20.0%)	0 (0.0%)	4 (14.3%)	6 (14.6%)	0.781
Two or more signs	0 (0.0%)	1 (33.3%)	8 (28.6%)	9 (22.0%)	0.117

Comparisons performed by Fisher’s exact tests.

**Table 4:** Surgical characteristics, time, and deaths by group.

	Initial symptomatic (10)	Late symptomatic (3)	Always asymptomatic (28)	Total (41)	*p*-value
Operated patients	9 (90.0%)	2 (66.7%)	15 (53.6%)	26 (63.4%)	0.114
Intraoperative rupture	2 (22.2%)	1 (50.0%)	1 (6.7%)	4 (15.4%)	0.171
Time between CT and surgery (days)					
Median (1^st^ Q, 3^rd^ Q)	0 (0, 3)	243 (6, 479)	15 (5, 42)	6 (2, 20)	0.001^K^
Minimum-maximum (n)	0-4 (9)	6-479 (2)	1-1961 (15)	0-1961 (26)	
Death at initial hospitalization	3 (30.0%)	0 (0.0%)	2 (7.4%)	5 (12.5%)	0.125
Hospitalization after CT	*	3 (100.0%)	15 (55.6%)	18 (60.0%)	0.215
Time of CT and last hospital register					
Median (1^st^ Q, 3^rd^ Q)	*	1216 (95, 1903)	346 (84, 1401)	373 (86, 1401)	0.610^M^
Minimum-maximum (n)	*	95-1903 (3)	0-3535 (26)	0-3535 (29)	

K: Kruskal-Wallis test. M: Mann-Whitney test. Other comparisons were performed using Fisher’s exact tests. 1^st^ Q: First quartile, 3^rd^ Q: Third quartile. *Not relevant data since they were treated at first.

**Table 5:** Characteristics of the second CT, and interval between the first (CT1) and second CTs (CT2).

	Initial symptomatic (10)	Late symptomatic (3)	Always asymptomatic (28)	Total (41)	*p*-value
New CT	0 (0.0%)	3 (100.0%)	8 (28.6%)	11 (26.8%)	0.003
CT2					
Median (1^st^ Q, 3^rd^ Q)		68 (30, 100)	58 (50, 74)	60 (50, 86)	0.776^M^
Minimum-maximum (n)		30-100 (3)	42-95 (8)	30-100 (11)	
Interval CT1 and CT2 (days)					
Median (1^st^ Q, 3^rd^ Q)		95 (5, 479)	373 (286, 835)	353 (95, 664)	0.279^M^
Minimum-maximum (n)		5-479 (3)	24-1961 (8)	5-1961 (11)	
Crescent sign in CT2		0 (0.0%)	2 (25.0%)	2 (18.2%)	>0.99
Focal wall discontinuity in CT2		2 (66.7%)	4 (50.0%)	6 (54.5%)	>0.99
Aortic bulges or blebs in CT2		0 (0.0%)	3 (37.5%)	3 (27.3%)	0.491
Draped aorta in CT2		0 (0.0%)	0 (0.0%)	0 (0.0%)	--
Rupture after CT2		3 (100.0%)	0 (0.0%)	3 (30.0%)	0.008

M: Mann-Whitney test. Other comparisons were performed using Fisher’s exact tests. 1^st^ Q: First quartile, 3^rd^ Q: Third quartile.
